# High-Risk Nonsecretory Multiple Myeloma With Poor Response to Daratumumab-Based Chemotherapy

**DOI:** 10.7759/cureus.78665

**Published:** 2025-02-07

**Authors:** Surendra Khanal, Rameela Rizvi, Christopher March

**Affiliations:** 1 Internal Medicine, Piedmont Athens Regional, Athens, USA

**Keywords:** clinical case report, daratumumab, lenalidomide, multiple myeloma prognosis, nonsecretory multiple myeloma, serum free light chain

## Abstract

Nonsecretory multiple myeloma (NSMM) is the variant of multiple myeloma defined by no detectable M protein secretion in serum or urine electrophoresis. Due to the lack of detectable M spike, diagnosis is often challenging and relies on bone marrow studies. As studies specifically focusing on NSMM are lacking, the current treatment guidelines are based on studies in secretory multiple myeloma. In this case report, we describe a 77-year-old female patient with a typical presentation of NSMM who did not respond to a chemotherapy regimen based on daratumumab.

## Introduction

Multiple myeloma is a malignant neoplasm of the plasma cells. It represents around 17% of hematologic malignancies with an estimated five-year survival rate of 61% ​[1].​ Cases are typically diagnosed between 65 and 74 years ​[2].​ Symptoms of multiple myeloma develop after infiltration of myeloma cells into organ systems or abnormal protein deposition in organs. Classically, symptoms are described as hypercalcemia, renal dysfunction, anemia, and bone pain (CRAB). There is an M protein spike in the serum and urine protein electrophoresis in patients with multiple myeloma which represents the abnormally increased protein secretion by the myeloma cells. However, in nonsecretory multiple myeloma (NSMM), the classic spike is not seen, making diagnosis more difficult. NSMM comprises about 3% of all myeloma cases. Multiple myeloma is confirmed by elevated plasma cells in bone marrow studies. Treatment is induction chemotherapy with or without autologous stem cell transplant followed by maintenance chemotherapy. The case highlights the challenges in the diagnosis and management of NSMM in situations where there is poor response to standard treatment regimens.

## Case presentation

A 77-year-old female patient presented to the emergency department with right-sided chest pain of two weeks duration. The pain was sharp and intermittent and was rated 10/10 in severity. She had similar but milder pain in the lower chest and left upper quadrant a few weeks prior to presentation which responded to over-the-counter analgesic medications. She had had ongoing weight loss for the few months prior to the presentation. Past medical history included chronic back and pelvic pain and a history of hairline rib fractures thought secondary to osteoporosis. Home medications were alendronate, calcium citrate, and cholecalciferol. She denied fever, chills, jaundice, easy bruising or bleeding, constipation, nausea, vomiting, or diarrhea. She denied any lower abdominal pain or dysuria. She was up to date with cancer screening for her age. She was a never-smoker with rare alcohol use. Family history was not significant for any hematologic malignancies. She was hemodynamically stable, with signs of mild dehydration, and had tenderness to palpation of the right lower chest wall and right upper quadrant. The rest of the examination was unremarkable. 

**Figure 1:**
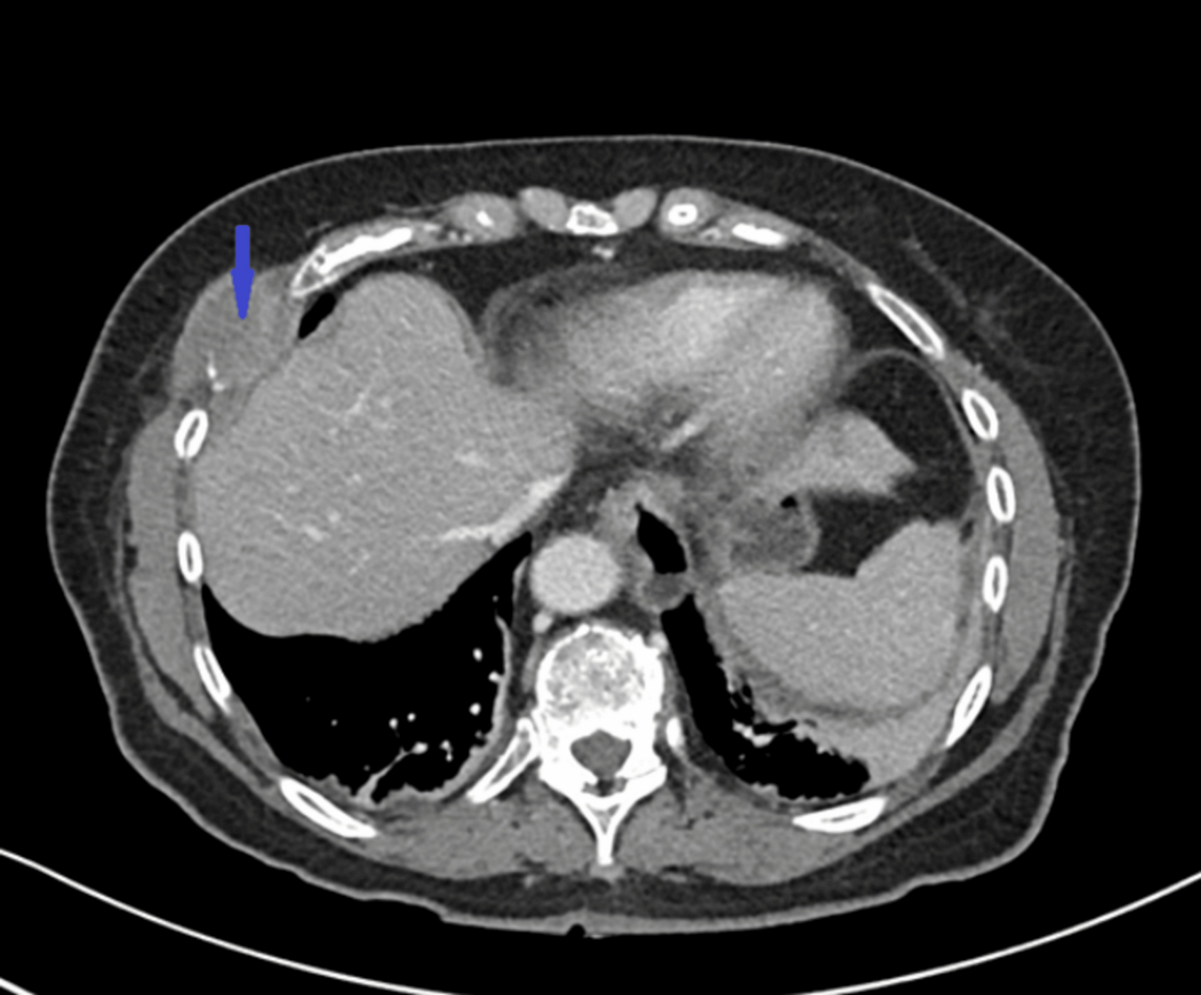
Cross-sectional CT image of the chest showing lytic lesion in the right sixth vertebrae (blue arrow)

**Figure 2:**
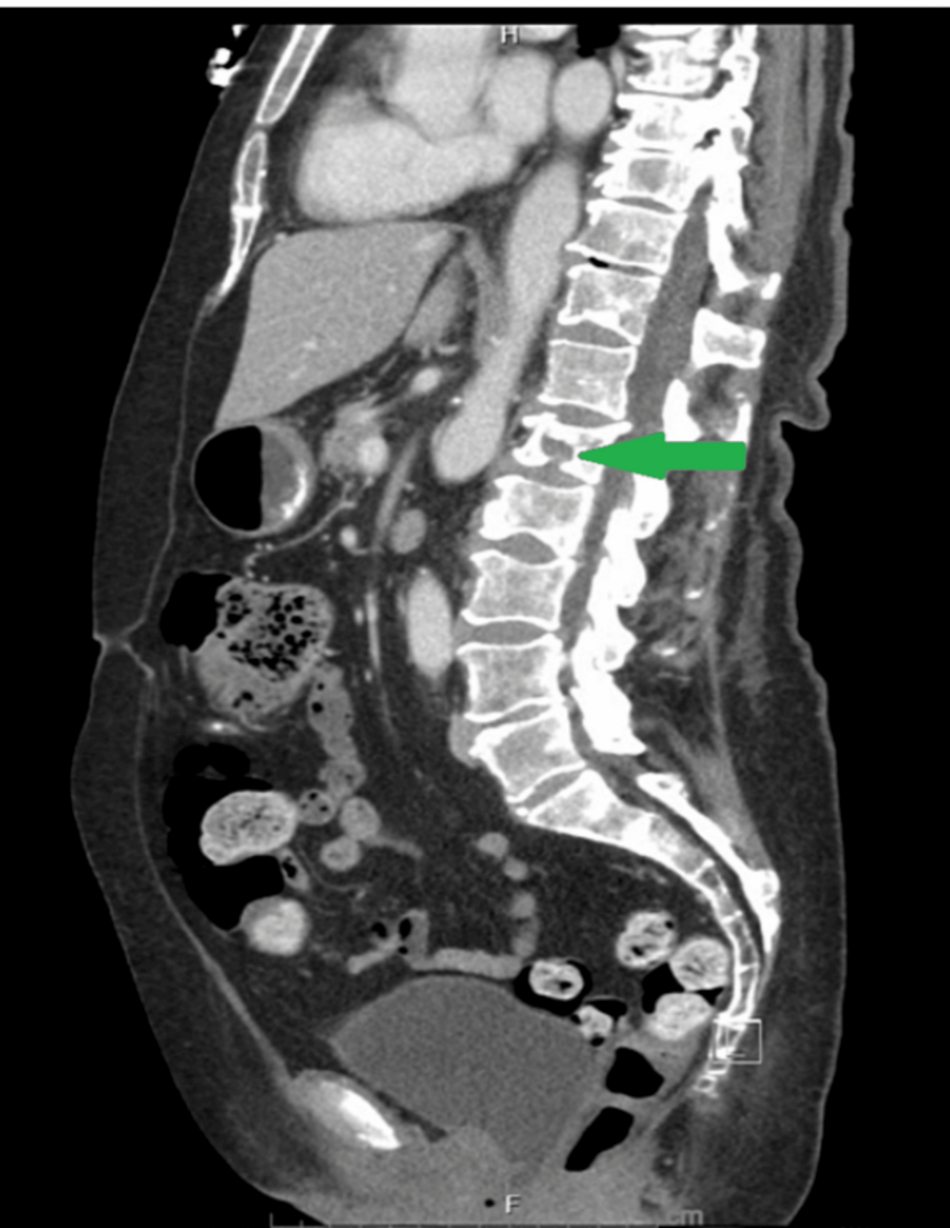
Sagittal CT section of the abdomen and showing L2 compression fracture due to multiple myeloma (green arrow)

Her initial labs were significant for renal dysfunction, anemia, and hypercalcemia meeting all the CRAB symptoms (Table 1). CT chest, abdomen, and pelvis were remarkable for diffuse destructive lesions involving the bilateral ribs, all the vertebrae, and pelvic bones. The largest lesion measured 3.5 cm x 2.2 cm in the anterior right sixth rib (Figure 1). There was a pathologic compression fracture on L2 with less than 25% height loss representing the severity of disease burden (Figure 2). 

**Table 1:** Initial laboratory blood workup revealing anemia, hypercalcemia, and elevated beta-2 microglobulin WBC: white blood cells; RBC: red blood cells; Hb: hemoglobin; MCV: mean corpuscular volume; GFR: glomerular filtration rate; PTH: parathyroid hormone

Blood tests	Result	Reference range
WBC	8.9	4.00-10.50 10^3^/µL
RBC	3.57	3.70-5.00 10^6^/µL
Hb	11.3	12.0-16.0 g/dL
MCV	95.6	78.0-100.0 fL
Platelets	131	130-400 10^3^/µL
Creatinine	1.84	0.40-1.00 mg/dL
GFR	28	>60 ml/min/m^2^
Calcium	13.4	8.4-10.2 mg/dL
PTH	12.90	15.00-80.00 pg/mL
Albumin	4.6	3.0-5.0 g/dL
Beta-2 microglobulin	6.21	<2.52 mg/L

The patient underwent a serum protein electrophoresis which was consistent with hypogammaglobulinemia with no detectable M spike contrasting the usual findings of secretory multiple myeloma (Table 2). Urine protein electrophoresis did not reveal any M spike or abnormally elevated proteins. Serum immunoglobin levels were consistent with hypogammaglobulinemia (Table 3). Serum-free light chain (SFLC) assay showed a ratio of 2.02, kappa 11.3, lambda 5.6. The combination of no M spike on electrophoresis and hypogammaglobulinemia suggested the diagnosis of NSMM.

**Table 2:** Serum protein electrophoresis shows no dominant protein secretion

Protein type	Result	Reference range
Total protein	5.9	6.1-8.1 g/dl
Alpha 1	0.4	0.2-0.3 g/dl
Alpha 2	1.1	0.5-0.9 g/dl
Beta 1	0.3	0.4-0.6 g/dl
Beta 2	0.2	0.2-0.5 g/dl
Gamma globulin	0.4	0.8-1.7 g/dl
Albumin	3.5	3.8-4.8 g/dl

**Table 3:** Immunoglobin levels on presentation show a low level consistent with hypogammaglobulinemia IgA: immunoglobulin A; IgG: immunoglobulin G; IgM: immunoglobulin M

Immunoglobulin type	Value	Reference range
IgA	20	70-320 mg/dL
IgG	363	600-1540 mg/dL
IgM	8	50-300 mg/dL

**Figure 3:**
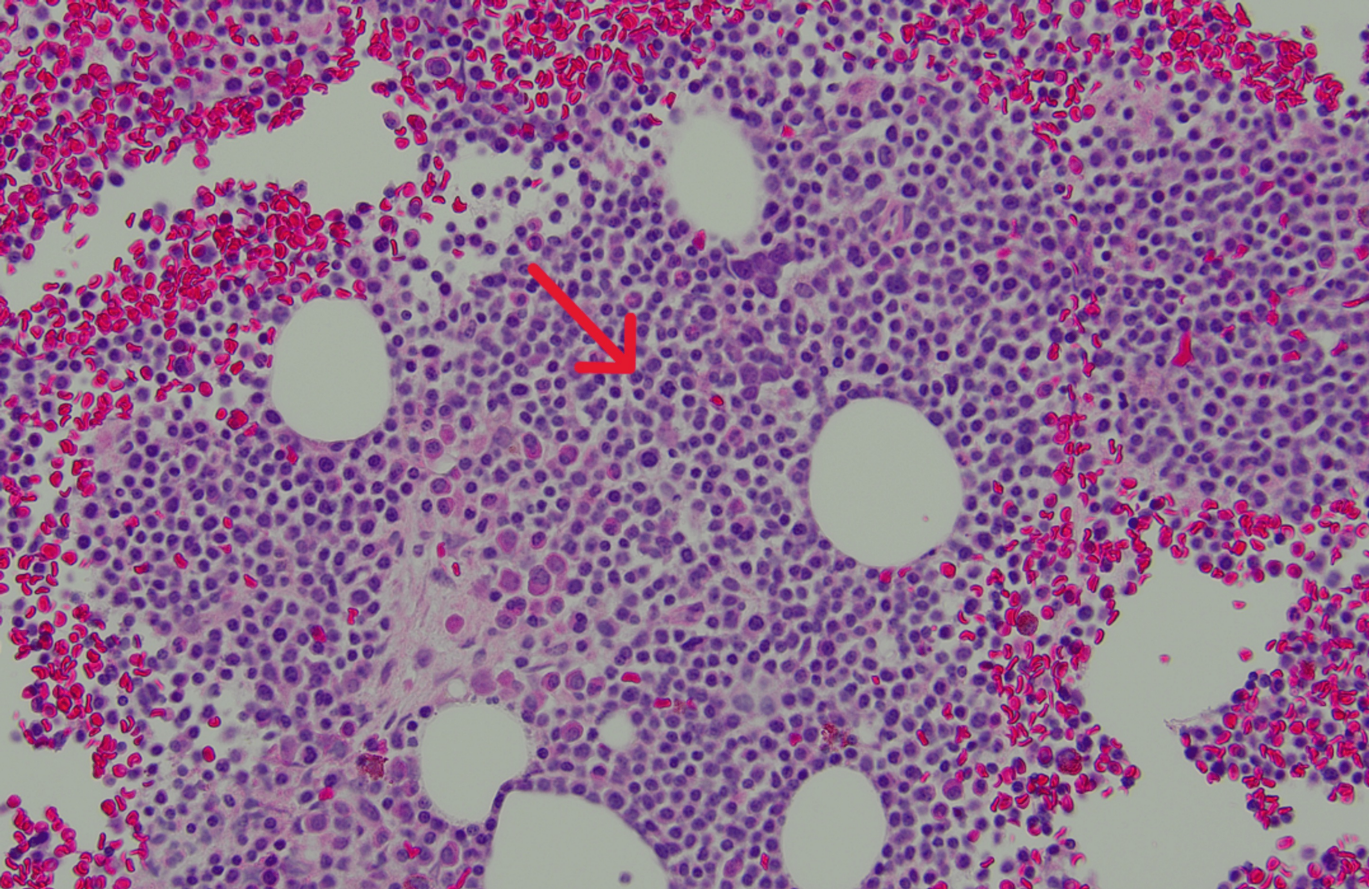
Hematoxylin and eosin stain of the bone marrow at 200X magnification showing sheets of plasma cells (red arrow)

**Figure 4:**
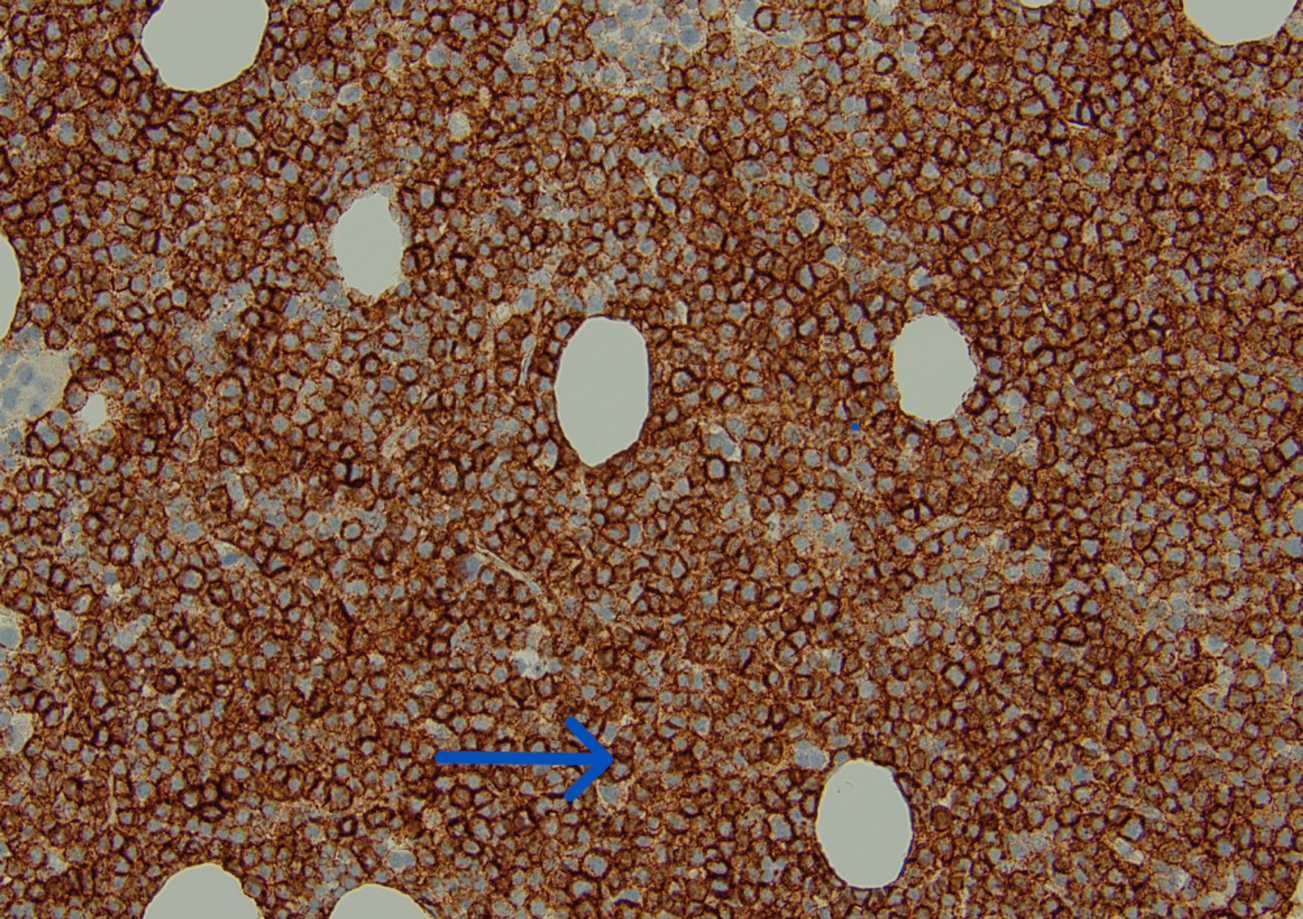
CD138 stain of bone marrow at 200x magnification highlighting plasma cells (blue arrow)

With a high suspicion of multiple myeloma, a CT-guided biopsy of the rib lesion on the right side was done. The biopsy result was positive for plasma cell neoplasm with 90% of the cells highlighted by a CD138 stain (Figures 3, 4). Flow cytometry revealed a plasma cell population, without any expression of surface and cytoplasmic light chain confirming NSMM. The myeloma fluorescence in situ hybridization (FISH) panel revealed IGH: CCND1(11;14) fusion, gain of 1q; loss of 1p, and monosomy 13. Cytogenetics showed a complex hypodiploid karyotype with t(11;14) (q13; q32.3) IGH: CCND1. Based on these findings, the patient was diagnosed with high-risk NSMM. 

She was started on palliative radiation therapy for the ribs and vertebrae as well as systematic chemotherapy with daratumumab, lenalidomide, and dexamethasone (D-Rd). Her disease progressed after four cycles of chemotherapy, and extensive metastasis to the C spine and skull base were found. Follow-up positron emission tomography-computed tomography (PET CT) showed multiple new lesions throughout the skeleton and pathological fractures in multiple ribs and the sacrum. Following the new findings, she chose to enroll in hospice, discontinuing aggressive treatment of her advanced disease.

## Discussion

Multiple myeloma is a rare hematologic malignancy with an overall prevalence of 2.1 per 100000 persons ​[3].​ Over the last two decades, there has been significant improvement in survival with the development of new drugs. Multiple myeloma develops from a premalignant hematologic condition called monoclonal gammopathy of undetermined significance (MGUS), an asymptomatic condition with a 3% prevalence in a population above 50 years of age ​[4,5].​ There is a classic M spike in the urine and serum electrophoresis in patients with multiple myeloma indicating a monoclonal protein secretion by tumor cells. Symptoms of multiple myeloma range from isolated bone lesions to fatal hyperviscosity syndrome with the most common presentation being bone pain and anemia; however, renal dysfunction and hypercalcemia are also frequently seen ​[6].​ All the symptoms are secondary to marrow replacement by malignant cells or abnormal protein deposition in extramedullary sites. Our patient presented with all the CRAB features; however, she had no evidence of monoclonal gammopathy.

NSMM only represents 3% of all myeloma cases ​[2].​ The prevalence of NSMM is more common in younger age with over 70% found in those under 65 years ​[7].​ As tumor cells do not secrete proteins, M spike is not seen on serum and urine electrophoresis, and diagnosis is often more challenging. SFLC assay is useful in diagnosing light chain-secreting multiple myeloma and can provide helpful prognostic information as well ​[8]. The current definition of NSMM includes all cases where M protein is not detected by serum or urine immunofixation ​[9]. This includes nonproducers, nonsecretors, and light chain multiple myeloma where the abnormal protein is not detectable by protein electrophoresis. Imaging can detect bone lesions and indicate tumor burden at the time of diagnosis. MRI and PET CT are superior to skeletal surveys in detecting tumor lesions ​[10].​ Our patient had normal electrophoresis and SFLC did not reveal an increase in light chains. The mild elevation of the kappa-to-lambda ratio could be explained by our patient's renal impairment, as both kappa and lambda were within the normal range. She had widespread bone disease at the time of diagnosis. The decision to pursue biopsy in our case was straightforward as our patient met all CRAB criteria; however, often in NSMM, the diagnosis is more challenging when one or more CRAB features are absent.

With the widespread availability of fluorescence in situ hybridization (FISH), cytogenetics studies have become a major part of myeloma workup. The presence of t(11,14) is usually seen in IgM, IgE, and NSMM and does not by itself confer a poor prognosis ​[11].​ The presence of translocations t(4;14), t(14,16), t(14,20), deletion del(17/17p), and gain (1q) in newly diagnosed multiple myeloma represents a high-risk disease implying poor prognosis with standard therapies ​[12]. ​This is regardless of the secretory nature of the disease. In fact, with modern-day treatment, nonsecretory myeloma may even have overall better survival outcomes compared to secretory myeloma ​[13].​ Our patient had a translocation involving t(11,14). In addition to being diagnosed at an older age, our patient had complex hypodiploid cytogenetics with a gain of 1q, indicating a high-risk feature and a poorer prognosis.

Given the rarity of NSMM, a detailed characterization of the disease is still lacking. Current treatment of NSMM is determined by the presence or absence of high-risk cytogenetic features which is similar to secretory multiple myeloma ​[9].​ Chemotherapy with autologous hematopoietic stem cell transplantation and consolidation, followed by maintenance therapy is the current standard of treatment. For patients ineligible for transplantation, a longer course of induction therapy is preferred. Daratumumab or bortezomib-based regimen with lenalidomide and dexamethasone is the current preferred regimen for multiple myeloma ​[14,15]. Our patient received four cycles of the D-Rd regimen. However, the disease progressed with the involvement of epidural space, and multiple new lytic lesions were seen on repeat imaging. Widespread disease on presentation and high-risk features are probably why our patient had such a poor response to the standard chemotherapy regimen. Given the fact that chemotherapeutic drugs are not readily tested on NSMM because of the difficulty of monitoring response, this represents an area of science yet to be explored. In similar cases where the response to D-Rd is poor, alternate regimens like proteasome inhibitors or second-line immunotherapies could be considered. 

Treatment monitoring is also quite difficult for NSMM compared to secretory myeloma. As tumor cells do not secrete immunoglobins, protein electrophoresis or immunofixation is not useful in monitoring response to therapy. When positive, the SFLC ratio can be used for monitoring. Imaging can guide the disease burden, and PET CT is preferred for follow-up in diagnosed cases of multiple myeloma ​[16].​ Finally, bone marrow studies can also be used for measuring disease burden and response to treatment ​[17].​ At the current level of understanding of NSMM, imaging and bone marrow studies should be the standard of treatment monitoring. Our patient underwent a follow-up PET CT that showed many new lesions with increased activity of the lesions. She also developed extensive skull base involvement along with cervical epidural metastasis. Although planned for repeat bone marrow biopsy, our patient decided on hospice, and monitoring response was deemed unnecessary.

## Conclusions

NSMM remains a diagnostic and therapeutic challenge requiring a higher degree of clinical suspicion. It is poorly understood in terms of clinical progression and response to standard chemotherapy. Current management guidelines are similar to secretory multiple myeloma which include daratumumab-based regimen. However, as reflected in the case, the response and prognosis of the disease to a standard chemotherapy regimen are not well-established. Monitoring of progression and response to treatment can be done using imaging and bone marrow biopsy. Studies focused on NSMM would be beneficial in further understanding this rare malignancy.
